# Study on Electrochemical Performance of MnO@rGO/Carbon Fabric-Based Wearable Supercapacitors

**DOI:** 10.3390/ma16134687

**Published:** 2023-06-29

**Authors:** Qianlan Ke, Yuhui Zhang, Yuanheng Fu, Chenxi Yang, Fan Wu, Zhongxiu Li, Yi Wei, Kun Zhang

**Affiliations:** 1 Key Laboratory of Textile Science & Technology (Ministry of Education), College of Textiles, Donghua University, Shanghai 201620, China; keql@dhu.edu.cn (Q.K.); zyh25611@163.com (Y.Z.); fyh000722@126.com (Y.F.); w13772247318@163.com (F.W.); jqlzx52580@163.com (Z.L.); 2 Shanghai High Performance Fibers and Composites Center (Province-Ministry Joint), Shanghai Key Laboratory of Lightweight Composite, Center for Civil Aviation Composites, Donghua University, 2999 North Renmin Road, Shanghai 201620, China; 3 State Key Laboratory for Modification of Chemical Fibers and Polymer Materials, College of Materials Science and Engineering, Donghua University, Shanghai 201620, China; chenxiyang0521@163.com

**Keywords:** hydrothermal, manganese oxide, graphene oxide, carbon fabric, supercapacitors

## Abstract

In this work, we reported the electrochemical performance of a type of carbon fabric-based supercapacitor by coating MnOx@rGO nanohybrids on carbon fabric with a simple one-step hydrothermal method. We studied the mass ratio of MnOx to rGO on the electrochemical properties of the carbon fabric-based supercapacitors. We found that as the mass ratio is 0.8:1 for MnO@rGO, the supercapacitor with a loading of 5.40 mg cm^−2^ of MnO@rGO nanohybrids on carbon fabric exhibits a specific capacitance of 831.25 mF cm^−2^ at 0.1 mA cm^−2^ current density. It also shows long-term cycling capacitance retention of 97.2% after 10,000 charge–discharge cycles at a current density of 0.4 mA cm^−2^. We speculate that the high electrochemical performance results from the strong interfacial bonding between the hierarchical architecture of MnO@rGO nanohybrids and carbon fabric.

## 1. Introduction

In recent years, wearable supercapacitors have attracted more and more attention due to their high power density, long cycle life, and environmental stability [1,2,3,4]. Generally, carbon, electrically conducting polymers, and metal oxides are the most widely used as active electrode materials in supercapacitors [5,6,7,8].

One major study on wearable supercapacitors is focused on the design and preparation of flexible carbon-based materials. Carbon fabrics are composed of a large number of carbon fibers possessing high surface area and an inter-fibers porous structure along with rough and absorptive surface properties [9,10]. Though carbon fabrics display poor electrochemical properties [11,12], they are still promising as an attractive alternative to flexible electrochemical electrodes owing to their light weight, good chemistry stability, high porosity, and flexibility [13,14,15]. In recent years, numerous researchers have led investigations on fabricating supercapacitors [16,17] by using carbon fabrics as both electrodes and mechanical support. Up to now, research has been concentrated on graphene-functionalized carbon fabric composites for supercapacitor applications. For example, Joaquín Artigas-Arnaudas et al. [18] reported structural supercapacitors fabricated from graphene nanoplatelets combined with PVDF and PVA binders on the carbon fiber fabrics surface with a mass-specific capacitance of 5.2 mF g^−1^ at 0.02 mA g^−1^ current density. Recently, Zehan Yao et al. [19] prepared flexible carbon fabric supercapacitors fabricated by vertical graphene and carbon fabric, with 13.2 mW cm^−2^ power density and 86.6 μWh cm^−2^ energy density. However, the relatively low energy density of carbon-based double-layer supercapacitors has limited their further practical applications in wearable supercapacitors [20]. Therefore, it is a new avenue to add transition metal oxides to carbon fabric-based supercapacitors.

Among all the metal oxides, manganese oxides (e.g., MnO, MnO_2_, and Mn_3_O_4_) exhibit excellent electrochemical activity owing to their broad potential window [21,22]. Compared to the widely reported MnO_2_ [23,24,25], manganese monoxide (MnO) has received less research attention, but has been one of the most promising active materials for electrochemical capacitors due to a high theoretical specific capacitance of ~1350 F g ^−1^, which is larger than that of MnO_2_ (1110 F g^−1^) [26,27]. Moreover, it possesses low cost and natural abundance and is environmentally friendly [28,29,30]. However, the lower electrical conductivity and cycling performance of MnO lead to its inferior rate capability, thereby limiting its further applications in developing high-performance MnO-based wearable supercapacitors [31,32].

At the same time, extensive efforts have been made to develop the hybridization of different types of graphene materials for supercapacitor applications, considering their high surface area, mass production, high electrical conductivity, and excellent chemical stability [33,34,35,36]. In order to improve the electrical conductivity of MnO/MnO_2_ electrodes, some plans that have attempted to induct conducting nanomaterials, such as graphene, have been adopted [37,38]. A. Gangwar et al. [39] prepared a type of nanocomposite (α-Mn_3_O_4_/MnO)@rGO via a urea-assisted sol–gel method calcined at 700 °C in an inert N_2_ atmosphere. The (α-Mn_3_O_4_/MnO)@rGO displayed an optimum specific energy of 10.8 Wh kg^−1^ and 3979 W kg^−1^ power density at 20 mA cm^−2^ current. Li et al. [40] fabricated free-standing flexible graphene/MnO_2_ composite papers (GMCP) by the preparation of GO/MnO_2_ dispersion, GO/MnO_2_ composite paper, as well as thermal reduction to generate GMCP. Although high performance can be achieved from the synthetic method, it still requires complicated steps with high energy consumption and low productivity.

In this work, the present investigation aimed at the incorporation of MnO@graphene sheet hybrids on carbon fabrics as novel and flexible electrode materials for supercapacitors. Manganese oxide/reduced graphene oxide coated on carbon fabric (MnO@rGO/C) was fabricated by a feasible route. The structural, morphological, and electromechanical properties were investigated. Interestingly, the as-prepared MnO@rGO@C electrodes with high specific capacitance and good cycle stability in 1 M Na_2_SO_4_ electrolyte make them a promising electrode material for supercapacitor applications.

## 2. Materials and Methods

### 2.1. Materials

The graphene oxide (GO) was bought from the Sixth ElementMaterials Technology Co., Ltd. (Changzhou, China). Potassium permanganate (KMnO_4_), anhydrous ethanol, and PVA 1788 (88% hydrolysis) were purchased from Shanghai Lingfeng Chemical Reagent Company (Shanghai, China). Manganese acetate tetrahydrate (Mn(CH_3_COO)_2_·4H_2_O) was purchased from J&K Scientific (Shanghai, China). The Na_2_SO_4_ was purchased from Shanghai Macklin Biochemical Company (Shanghai, China). Silver paste was purchased from the Shenzhen Sunflower Electronic Company (Shenzhen, China). The PTFE filament was purchased from Shantou Mingda Textile Company (Shantou, China). These materials were used as received without further purification or treatment.

### 2.2. Preparation of MnOx@GO Slurry

The MnOx@GO slurry was prepared via a simple, one-step hydrothermal method: Firstly, 15 mL of GO suspension (0.5 mmol) was subjected to ultrasonic vibration for 30 min. The Mn(CH_3_COO)_2_·4H_2_O (0.025 mmol, 20 mL) was added to the GO solution and then underwent further ultrasonic mixing for 30 min. It was then mixed with KMnO_4_ (0.2 mmol, 10 mL) and stirred at 85 °C for 1 h. The MnO_x_@GO slurry was concentrated after washing with water using vacuum filtration several times. The mass ratio of MnO_x_ to GO in MnO_x_@GO composites was controlled to be 0:1, 0.5:1, 0.8:1, and 1:1 by changing the molar concentrations of Mn(CH_3_COO)_2_·4H_2_O and KMnO_4_.

### 2.3. Preparation of MnOx@rGO/C

MnO_x_@rGO/C was prepared by the following three-step method. First, the above MnO_x_@GO slurry was thermally concentrated to a suitable concentration of 5 mM. Then, the as-received carbon fabric was immersed into the condensed MnOx@GO slurry and dried in a vacuum oven. The procedure was repeated 6–7 times. Finally, the MnO_x_@rGO/C sample was obtained by carbonizing the MnO_x_@GO/C in a high-temperature vacuum tubular furnace (OTF-1200X) at 800 °C for 3 h under an Argon (Ar) atmosphere with a heating rate of 10 °C/min. In comparison, the as-received carbon fabric was named C.

### 2.4. Preparation of MnO_x_@rGO/C Electrodes and Supercapacitors

One piece of MnO_x_@rGO/C with the mass ratio of MnO_x_ to GO of 0:1, 0.5:1, 0.8:1, and 1:1 with the area of 1 cm × 1.1 cm was fixed on the plastic chuck to form the working electrode and soaked in 1 ml 1 M Na_2_SO_4_ aqueous electrolyte. The all-solid-state MnO_x_@rGO_(0.8:1)_/C supercapacitors were prepared as described next. One gram of PVA powder was added to the mixture of 9 mL of DI water. Then, the mixture was heated to 90 °C under vigorous stirring for 30 min until the solution was cooled down to room temperature. Then, 1.42 g of Na_2_SO_4_ crystalline particles were added to the mixture of 1 mL of DI water. Following this, 1 mol Na_2_SO_4_ solution was added to the above PVA solution and stirred thoroughly to prepare the PVA/Na_2_SO_4_ gel electrolyte. The assembled MnO_x_@rGO/C supercapacitors consist of two MnO_x_@rGO/C electrodes in parallel with the PVA/Na_2_SO_4_ gel electrolyte in between.

### 2.5. Characterizations

A scanning electron microscope (SEM, HITACHI, TM3000 and SU5000) and transmission electron microscope (TEM, JEOL, JEM-2100) were used to characterize the surface morphologies of the electrodes. The characteristic elements were detected using an energy-dispersive X-ray spectrometer (EDS, Bruker, Billerica, MA, USA). Raman spectroscopy (Renishaw Micro-Raman/Photoluminescence System) was used to analyze the carbon structure of as-produced GO/C, MnO_x_@rGO/C and reduced MnO_x_@rGO/C using a 633 nm He-Ne laser. The XRD patterns of samples were obtained with an X-ray diffractometer with Cu Ka radiation (Υ = 1.54056° A), (D/Max-2550 PC, Rigaku, Tokyo, Japan). X-ray photoelectron spectroscopy (XPS, ESCALAB250Xi) was used to characterize the element valence states and contents. The four-probe method (Scientific Equipment & Services) was used to measure the electrical conductivity of C, rGO@C, and MnO/rGO@C samples.

### 2.6. Characterizations of Electrochemical Performances

Electrochemical performance of C, rGO/C, and MnO_x_@rGO/C electrodes, including cyclic voltammetry (CV) tests, galvanostatic charge/discharge (GCD) tests, and electrochemical impedance spectra (EIS, 0.01 Hz to 100 kHz), were carried out on an electrochemical workstation (CHI 660E, CH Instruments Inc., Shanghai, China)with a three-electrode cell that contained a working electrode, a counter electrode (Pt foil), and a reference electrode (Ag/AgCl electrode). CV and GCD curves of the electrode were performed in a potential window of 0 to 0.8 V and the area capacitance of the supercapacitor (C_A_) was calculated from GCD curves at different current densities.

## 3. Results and Discussion

**Figure 1:**
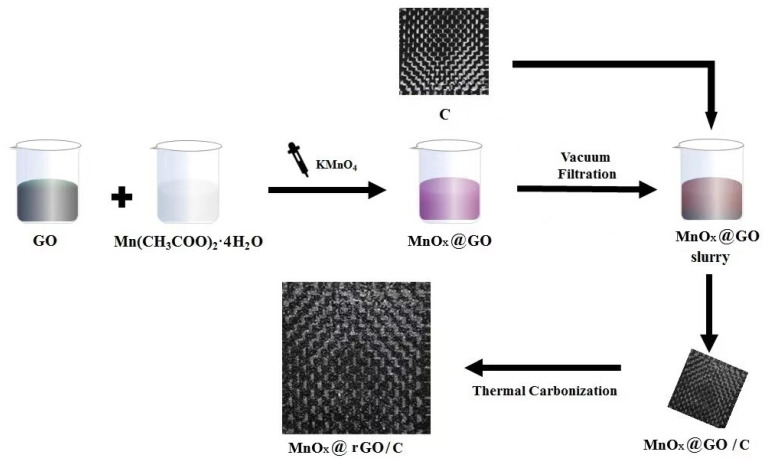
Schematic illustration for the preparation of MnO_x_@rGO/C.

The synthesis roadmap of MnO_x_@rGO/C was shown in Figure 1. Briefly, a MnO_x_@GO slurry was prepared using a one-step hydrothermal method, where MnO_x_ nanoflakes were grown in situ on GO sheets, forming tight interactions. Next, C was repeatedly immersed in the MnO_x_@GO slurry. After drying, MnO_x_@GO/C was obtained. Finally, MnO_x_@rGO/C was formed by thermal carbonization under Ar purge for heat reduction of GO to rGO. For electrochemical measurements, one piece of MnO_x_@rGO/C electrode was immersed into Na_2_SO_4_ aqueous electrolyte.

**Figure 2:**
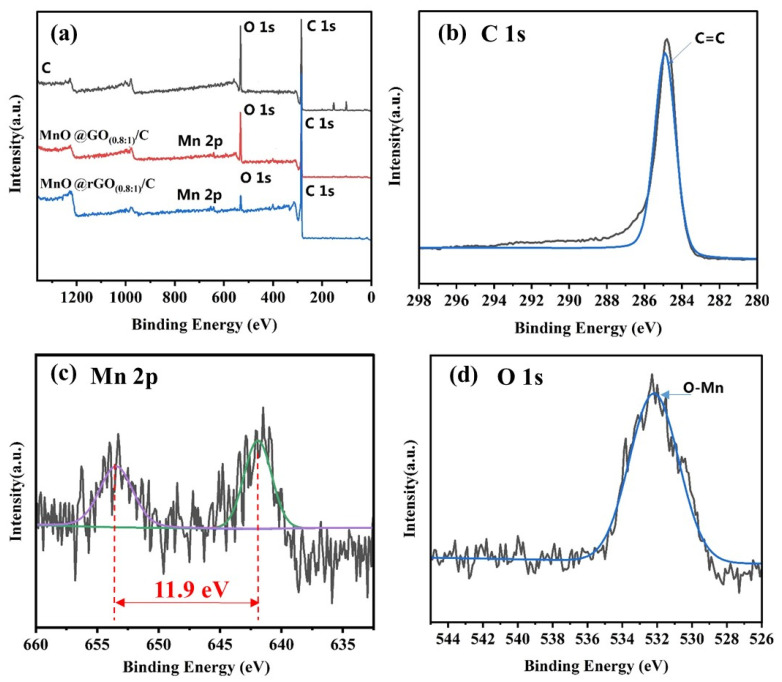
(**a**) XPS survey of C, MnO_x_@GO_(0.8:1)_/C, and MnO_x_@rGO_(0.8:1)_/C; (**b**) XPS C 1s, (**c**) XPS for Mn 2p; different colours in subfigure can be attributed to the peaks of Mn 2p3/2 and Mn 2p1/2 and are located at 642 eV and 653.9 eV, respectively, and (**d**) O1s spectra of MnO_x_@rGO_(0.8:1)_/C.

The compositional analysis of the C, MnO_x_@GO_(0.8:1)_/C, and MnO_x_@rGO_(0.8:1)_/C was characterized by XPS and the corresponding results are presented in Figure 2. As shown in Figure 2a, the XPS spectrum of C only presents two elements, namely, C and O. The sample of MnO_x_@GO_(0.8:1)_/C and MnO_x_@rGO_(0.8:1)_/C contains Mn, C, and O elements, proving the successful deposition of MnO on C. In contrast, the signal of manganese (Mn 2p) emerges in the XPS survey spectrum of MnO_x_@GO_(0.8:1)_/C and MnO_x_@rGO_(0.8:1)_/C, illustrating the attachment of MnO_x_ on the surface of rGO sheets [41]. Figure 2b shows the C 1s spectrum of MnO_x_@rGO_(0.8:1)_/C; the spectrum reveals the presence of C=C (284.9 eV), which is consistent with the literature data [42]. The Mn 2p spectrum of MnO_x_@rGO_(0.8:1)_/C (Figure 2c) shows that the peaks of Mn 2p_3/2_ and Mn 2p_1/2_ are located at 642 eV and 653.9 eV, respectively, with an energy separation of 11.9 eV, which is in good agreement with the reported literature data of Mn 2p_3/2_ and Mn 2p_1/2_ in MnO [31]. Figure 2d reveals the O 1s spectrum of MnO_x_@rGO_(0.8:1)_/C, while the peaks located at 532 eV reveal the presence of an O-Mn bond [43]. In summary, the above XPS results further demonstrated the successful product of MnO.

**Figure 3:**
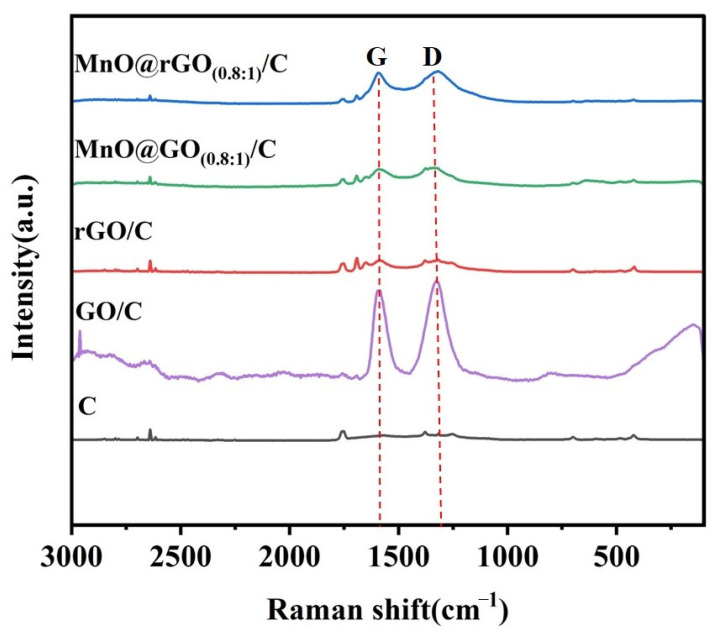
Raman spectra for C, GO/C, rGO/C, MnO@GO_(0.8:1)_/C, and MnO@rGO_(0.8:1)_/C.

Next, Raman spectroscopy was utilized to investigate the graphitization and defect degree of the as-prepared sample. Figure 3 shows the Raman spectra of C, GO/C, rGO/C, MnO@GO_(0.8:1)_/C, and MnO@rGO_(0.8:1)_/C. The G band is associated to the highly ordered graphite of the samples. For GO/C, the G band at 1585 cm^−1^ is characteristic of sp^2^-hybridized C–C bonds [44]. The D band is attributed to the structural defects of the samples. Meanwhile, the D band located at 1329 cm^−1^ is corresponding to the defects and disorder carbon in the graphite layers [45]. The I_D_/I_G_ values of GO/C, rGO/C, MnO@GO_(0.8:1)_/C, and MnO@rGO_(0.8:1)_/C are 1.016, 0.970, 1.074, and 1.054, respectively. Obviously, the defect degree of carbon materials in rGO/C is lower than that of GO/C. Additionally, the I_D_/I_G_ values of MnO@GO_(0.8:1)_/C are larger than that of MnO@rGO_(0.8:1)_/C, and the adhesion between MnO and C in MnO@rGO_(0.8:1)_/C is relatively more tight than that in MnO@GO_(0.8:1)_/C.

**Figure 4:**
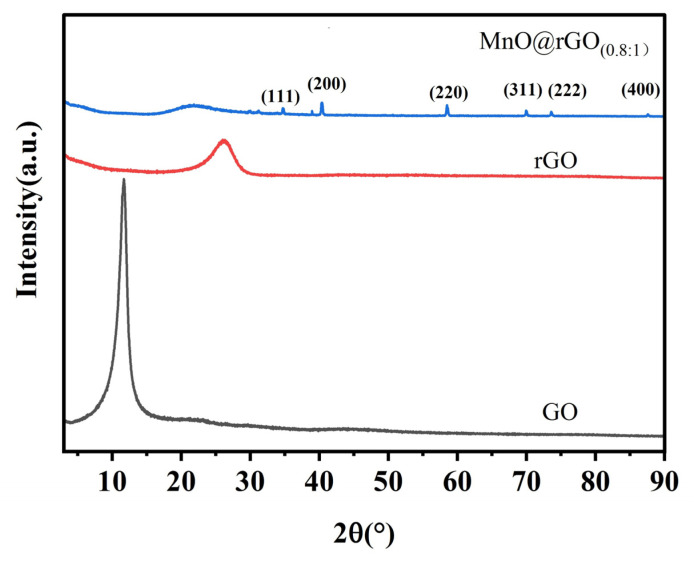
XRD patterns for GO, rGO, and MnO@rGO_(0.8:1)_.

Figure 4 depicts the XRD spectra of GO, rGO, and MnO@rGO_(0.8:1)_. The X-ray diffraction spectrum of GO reflects a characteristic peak at 2θ = 11.4°, corresponding to (002) crystal plane of graphite [46]. In the case of rGO, a characteristic broad peak around 2θ = 24.5° was observed, which corresponds to the (002) plane of the graphite structure [47]. The diffraction peaks of MnO@rGO_(0.8:1)_ at 2θ of 34.9°, 40.5°, 58.5°, 70.2°, 74°, and 88° correspond to the (111), (200), (220), (311), (222), and (400) lattice planes, respectively, indicating the presence of MnO nanocrystals (JCPDs no. 07-0230) [26,31]. The peak shift of rGO between rGO and MnO@rGO can be attributed to the reduced space between rGO layers.

**Figure 5:**
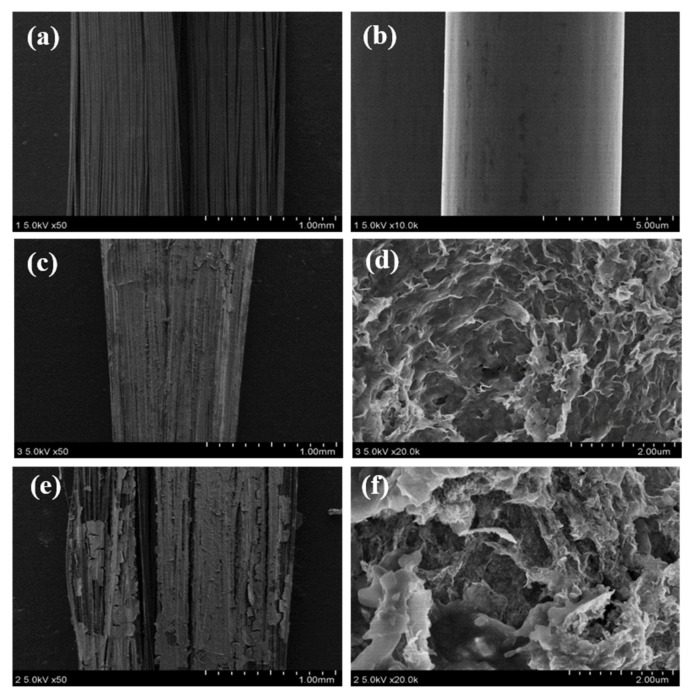
Typical SEM images: (**a**) low-resolution (LR) view of C; (**b**) high-resolution (HR) view of C; (**c**) LR view of MnO@GO_(0.8:1)_/C; (**d**) HR view of MnO@GO_(0.8:1)_/C; (**e**) LR view of MnO@rGO_(0.8:1)_/C; (**f**) HR view of MnO@rGO_(0.8:1)_/C.

The SEM images of C, MnO@GO_(0.8:1)_/C, and MnO@rGO_(0.8:1)_/C are shown in Figure 5a–e, respectively. Figure 5a,b shows the SEM images of C without any attached MnO@GO nanoparticles, while MnO@GO_(0.8:1)_/C (Figure 5c) and MnO@rGO_(0.8:1)_/C (Figure 5e) possess rougher surfaces compared to that of C. Moreover, GO nanosheets with clean and wrinkled surfaces [48] can be seen in Figure 5d. Evenly distributed rGO [49] were well-connected on the surface of MnO@rGO_(0.8:1)_/C, as shown in Figure 5f. More MnO nanoparticles uniformly gathered and grew in situ on the surface of C with the aid of rGO [26].

**Figure 6:**
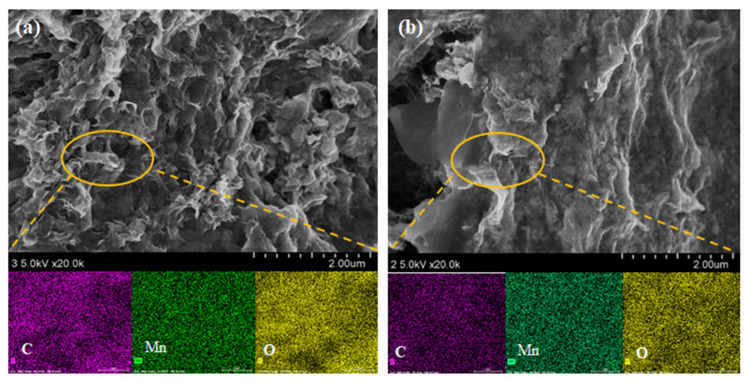
(**a**) EDS mapping for C, Mn, O elements of MnO@GO_(0.8:1)_/C and (**b**) EDS mapping for C, Mn, and O elements of MnO@rGO_(0.8:1)_/C.

Elemental mapping by EDS was used to illustrate the distribution of C, Mn, and O elements in MnO@GO_(0.8:1)_/C and MnO@rGO_(0.8:1)_/C, respectively, as shown in Figure 6a,b. Figure 6a shows that Mn is distributed homogeneously throughout the sample, implying the successful synthesis of MnO on the MnO@GO_(0.8:1)_/C. The elemental compositions of MnO@GO_(0.8:1)_/C were 12.92 wt%, 51.26 wt%, and 26.70 wt% for C, Mn, and O, respectively. The elemental compositions of MnO@rGO_(0.8:1)_/C were 10.77 wt%, 74.62 wt%, and 13.29 wt% for C, Mn, and O, respectively.

**Figure 7:**
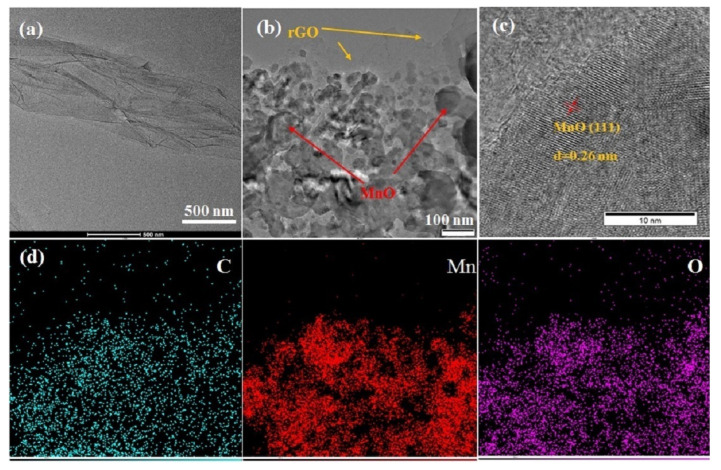
(**a**) LR TEM image of GO; (**b**) LR TEM image of MnO@rGO_(0.8:1)_; (**c**) HR TEM image of MnO@rGO_(0.8:1)_; (**d**) EDS mapping for C, Mn, and O elements of MnO@rGO_(0.8:1)_/C.

We used TEM characterizations to further verify the structure of MnO@rGO_(0.8:1)_/C. Under the low magnification of GO, Figure 7a illustrates multi-layered wrinkled GO sheets, while wrinkles seem to have disappeared in rGO, as shown in Figure 7b. The high-resolution TEM image (Figure 7c) shows the periodic lattice fringe with an interplanar distance of about 0.26 nm, corresponding to the MnO (111) face [43]. It confirms the nanoflake morphology of MnO in MnO@rGO_(0.8:1)_/C. In addition, the element mapping (Figure 7d) shows an even distribution of C, Mn, and O elements in MnO@rGO_(0.8:1)_/C.

**Figure 8:**
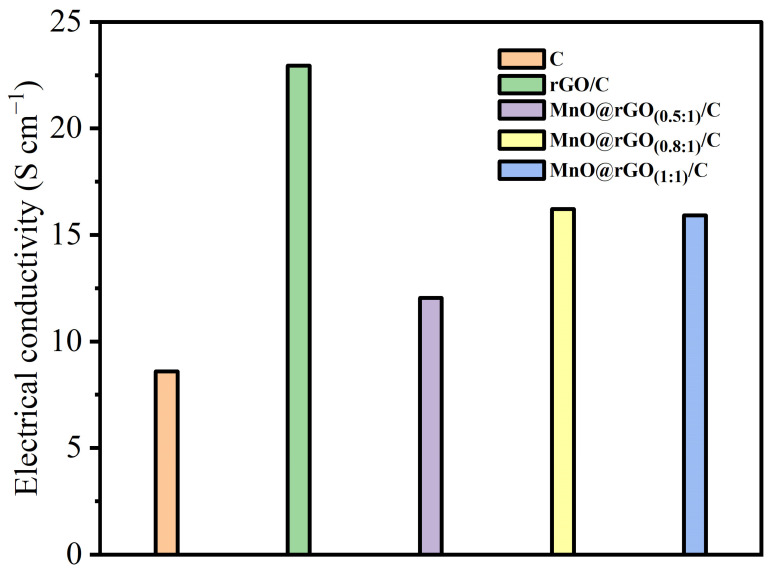
Electrical conductivities of C, rGO/C, and MnO@rGO/C electrodes with different mass rations of MnO to rGO at a small mass loading of active material (1.08 mg cm^−2^).

Furthermore, CV and GCD experiments were performed to investigate the electrochemical behavior of the C, rGO/C, MnO@rGO_(0.5:1)_/C, MnO@rGO_(0.8:1)_/C, and MnO@rGO _(1:1)_/C samples. It can be seen from Figure 8 that the electrical conductivities of C, rGO/C, MnO@rGO_(0.5:1)_/C, MnO@rGO_(0.8:1)_/C, and MnO@rGO_(1:1)_/C are 8.6 S cm^−1^, 22.95 S cm^−1^, 12.05 S cm^−1^, 16.22 S cm^−1^, and 15.92 S cm^−1^, respectively. The decreased conductivity of MnO@rGO/C is due to the semiconductor properties of MnO that lead to poor electrical transport. In contrast, the largest electrical conductivity is rGO/C, due to the stacking of the high-conductivity rGO layer on C.

**Figure 9:**
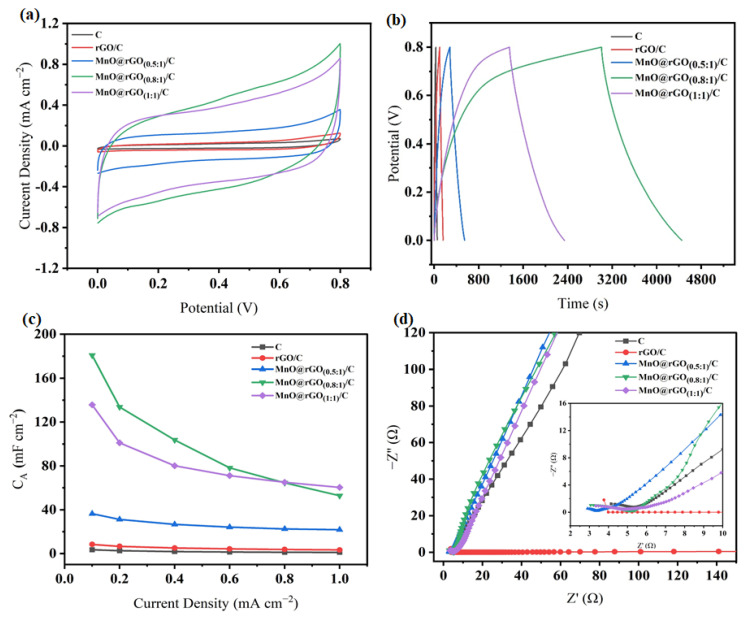
Electrochemical performances of C, rGO/C, and MnO@GO/C electrodes with different mass ratios of MnO to rGO at a small mass loading of active material (1.08 mg cm^−2^) tested in a three-electrode cell with 1M Na_2_SO_4_ aqueous electrolyte. (**a**) CV curves recorded at a scan rate of 5 mV s^−1^; (**b**) GCD curves measured at a current density of 0.1 mA cm^−2^; (**c**) areal specific capacitances (C_A_) based on GCD test and measured at different current densities; (**d**) Nyquist plots of C, rGO/C, and MnO@rGO/C electrodes.

The electrochemical performance as a function of the mass ratio of MnO to rGO in MnO@rGO/C composites at relatively low mass loading of active material (1.08 mg cm^−2^) is illustrated in Figure 9a–d. The typical CV curves of the prepared electrodes at 5 mV s^−1^ scan rate are presented in Figure 9a. Obviously, all the shapes of CV curves demonstrate near-rectangular shapes, which indicates good symmetrical reversible electrochemical reactions. Meanwhile, the CV curves of the MnO@rGO/C electrodes show indistinct redox peaks within the working voltage range, which may be caused by the electrical double layer charge storage of rGO, along with fast surface redox reactions between Na^+^ from Na_2_SO_4_ electrolyte and MnO nanoflakes [50]. The areas of CV curves of MnO@rGO_(0.5:1)_/C, MnO@rGO_(0.8:1)_/C, and MnO@rGO_(1:1)_/C samples are much larger than those of C and rGO/C. It indicates that the introduction of MnO significantly improves the electrochemical performance of flexible carbon fabrics. With the increase of MnO content, the areas of the CV curves of MnO@rGO/C electrodes show an enlarged tendency, reaching the maximum peak in the MnO@rGO_(0.8:1)_/C electrode. However, as the content of MnO continues to increase, it leads to diminished areas of CV curves.

GCD curves of the C, rGO/C, MnO@rGO_(0.5:1)_/C, MnO@rGO_(0.8:1)_/C, and MnO@rGO_(1:1)_/C electrodes have good charge–discharge performance and share nearly symmetrical triangular shapes, suggesting a high reversibility. The charge–discharge curves of C, rGO/C, and MnO@rGO/C electrode materials are approximately symmetrical, but the symmetry of their triangular charge–discharge curves becomes worse after the electrode materials are loaded with MnO. Moreover, the discharge time for MnO@rGO_(0.8:1)_/C is 1446 s, which is the longest of all the electrodes. IR drops are detectable in MnO@rGO/C electrodes, as low as 1.7 mV for the MnO@rGO_(0.8:1)_/C electrode, which is much smaller than that of the C electrode (21.3 mV), rGO/C electrode (11.3 mV), and for MnO@rGO_(0.5:1)_/C (2.8 mV) and 2 mV for MnO@rGO_(1:1)_/C (2 mV), respectively. The areal capacitance (C_A_) value calculated based on the GCD curve reaches a maximum of 180.75 mF cm^−2^ for th4 MnO@rGO_(0.8:1)_/C electrode, which is twenty-one times that of the rGO/C electrode (8.35 mF cm^−2^). Both the low C_A_ value and high IR drop of the rGO electrode may result from the stacking of rGO sheets, which impedes charge transfer and ion diffusion in rGO. However, for MnO@rGO_(0.8:1)_/C, MnO nanoflakes are well-distributed on rGO, which increases the spaces between rGO sheets, effectively alleviating the stacking problem and improving the charge storage ability.

The C_A_ calculated from discharge curves is shown in Figure 9c. The maximum areal specific capacitances of the C, rGO/C, MnO@rGO_(0.5:1)_/C, MnO@rGO_(0.8:1)_/C and MnO@rGO _(1:1)_/C were 3.45 mF cm^−2^, 8.35 mF cm^−2^, 36.42 mF cm^−2^, 180.75 mF cm^−2^, and 135.71 mF cm^−2^, respectively. The MnO@rGO_(0.8:1)_/C has a better area-specific capacitance than the other samples. This is believed to be caused by MnO on the carbon fiber completely covering the carbon fiber, forming a nanosheet structure that increases the specific surface area, thereby improving the electrode performance to collect electrons and increasing C_A_.

The electrochemical behavior of the C, rGO/C, MnO@rGO_(0.5:1)_/C, MnO@rGO_(0.8:1)_/C, and MnO@rGO_(1:1)_/C supercapacitors was further characterized by EIS. At a high frequency, the electron transfer limited avenue can be reflected, while at a low frequency, the diffusion process is displayed [51]. In Figure 9d, the MnO@rGO_(0.8:1)_/C displays a higher slope in the low-frequency region, which suggests that the MnO@rGO_(0.8:1)_/C has ideal charge storage capacitive behavior compared to that of the rGO/C, MnO@rGO_(0.5:1)_/C, MnO@rGO_(0.8:1)_/C, and MnO@rGO_(1:1)_/C. A close-up observation of the high-frequency region of the Nyquist plots revealed a semicircle with a small diameter followed by a short 45° Warburg region, indicating low charge transfer resistance and fast ion diffusion within the MnO@rGO/C electrodes, which could be attributed to the high electrical conductivity and unique structure of MnO@rGO/C.

**Figure 10:**
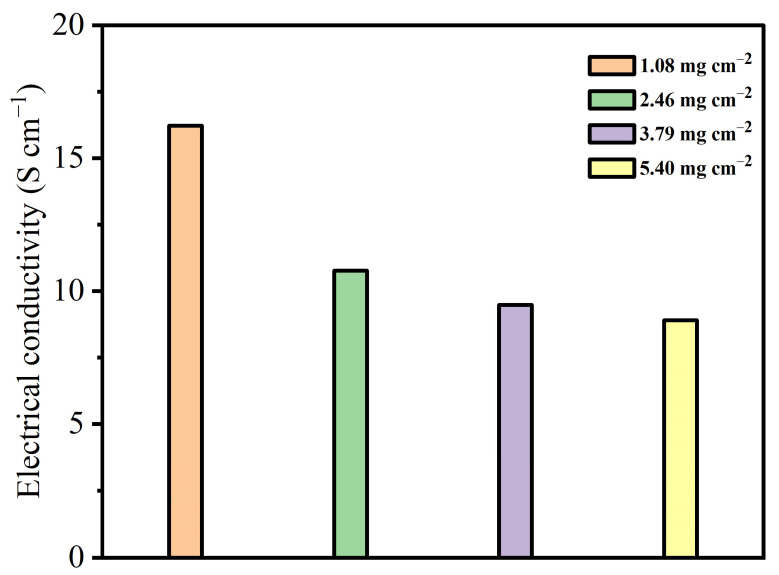
Electrical conductivities of MnO@rGO_(0.8:1)_/C electrodes at different mass loadings of active material (1.08 mg cm^−2^–5.40 mg cm^−2^).

Furthermore, as shown in Figure 10, the electrical conductivity of MnO@rGO_(0.8:1)_/C electrodes at different mass loadings of active material (1.08 mg cm^−2^–5.40 mg cm^−2^) is 16.22 S cm^−1^, 10.77 S cm^−1^, 9.48 S cm^−1^, 8.90 S cm^−1^, respectively. The decreasing conductivity of MnO@rGO/C is due to the semiconductor properties of manganese oxide, resulting in poor electrical transport in electrodes.

**Figure 11:**
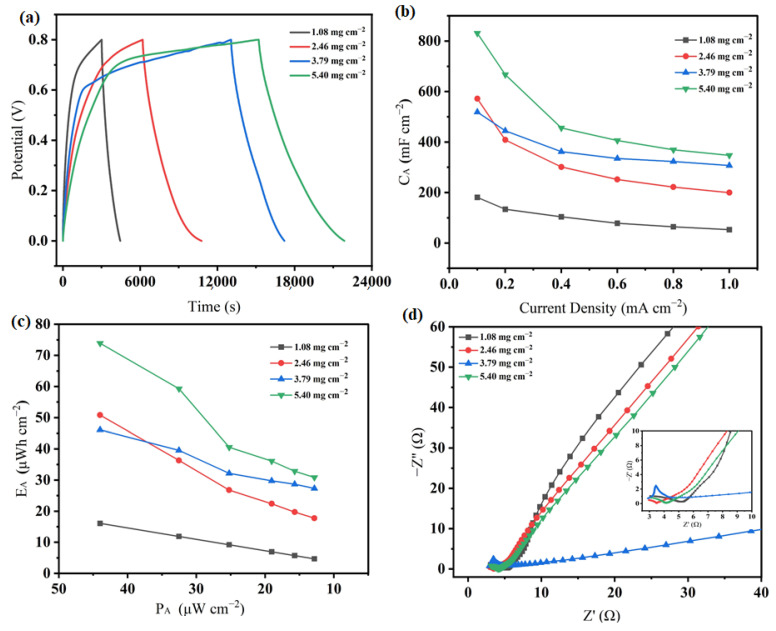
Electrochemical performances of MnO@rGO_(0.8:1)_/C at different mass loadings of active material (1.08 mg cm^−2^–5.40 mg cm^−2^). (**a**) GCD curves of MnO@rGO_(0.8:1)_/C measured at a current density of 0.1 mA cm^−2^; (**b**) area-specific capacitances(CA) of MnO@rGO_(0.8:1)_/C based on GCD test and measured at different current density; (**c**) Ragone plots of MnO@rGO_(0.8:1)_/C electrodes; (**d**) Nyquist plots of MnO@rGO_(0.8:1)_/C.

We then analyzed the effect of the mass loading of active material (MnO@rGO_(0.8:1)_) on the electrochemical performance of MnO@rGO_(0.8:1)_/C electrodes (1.08 mg cm^−2^–5.40 mg cm^−2^, Figure 11). From the near-symmetrical GCD curves (Figure 11a), it indicates that all MnO@rGO_(0.8:1)_/C electrodes possess electrochemical reversibility as well as excellent pseudo-capacitive characteristics.

More importantly, as shown in Figure 11b, the areal specific capacitances of the MnO@rGO_(0.8:1)_/C (1.08 mg cm^−2^–5.40 mg cm^−2^) are 180.75 mF cm^−2^, 571.75 mF cm^−2^, 518.75 mF cm^−2^, and 831.25 mF cm^−2^, respectively, suggesting that MnO@rGO_(0.8:1)_/C (5.40 mg cm^−2^) has the highest C_A_ among all samples. It is believed to be caused by the high loading of MnO@rGO nanohybrids on the carbon fiber, forming a continuous nanostructure that increases the specific surface area. Therefore, it can enhance the electrode’s ability to store more charges and electrons, thus increasing the area-specific capacitance. Manganese oxide has poor conductivity, which can lead to a low coulombic effect. The coulombic efficiency of the MnO@rGO_(0.8:1)_/C at 5.40 mg cm^−2^ mass loadings of active material was 0.44. This may be attributed to rGO covered by MnO, which hinders the penetration of electrolyte ions into the electrodes.

Ragone plots for MnO@rGO_(0.8:1)_/C electrodes at different mass loadings of active material (1.08 mg cm^−2^–5.40 mg cm^−2^) are shown in Figure 11c. The MnO@rGO_(0.8:1)_/C (5.40 mg cm^−2^) had the largest E_A_ (73.9 μWh cm^−2^), higher than that of MnO@rGO_(0.8:1)_/C (1.08 mg cm^−2^) (16.07 μWh cm^−2^).

In addition, the EIS slope of the MnO@rGO_(0.8:1)_/C electrode (5.40 mg cm^−2^) at low frequency is close to 45° (Figure 11d). This inference is confirmed by Nyquist plots of MnO@rGO_(0.8:1)_/C electrodes with different mass loadings. When the mass loading of MnO@rGO_(0.8:1)_/C increases from 1.08 mg cm^−2^ to 5.40 mg cm^−2^, the ESR values were 3.06 Ω, 2.97 Ω, 3.30 Ω, and 3.13 Ω, respectively, and the slope at low frequency remains stable. In addition, overloaded MnO@rGO_(0.8:1)_/C (5.40 mg cm^−2^) may have a negative effect on ion transport and storage due to the decreased ion-accessible surface area caused by the dense structure. For the MnO@rGO_(0.8:1)_/C electrode (2.46 mg cm^−2^), it shows the smallest ESR value.

**Figure 12:**
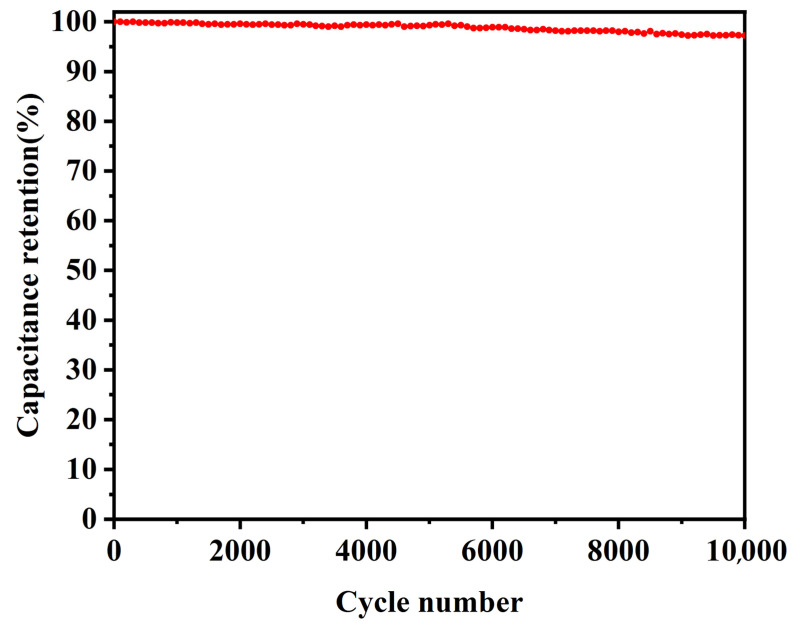
Cycle life of MnO@rGO_(0.8:1)_/C at a mass loading 5.40 mg cm^−2^.

Additionally, cycling stability is also a vital performance index for supercapacitors. Figure 12 shows that the MnO@rGO_(0.8:1)_/C (5.40 mg cm^−2^) electrode has a long-term cycling stability of 97.2% capacitance retention after 10,000 charge–discharge cycles at 0.4 mA cm^−2^ current density.

**Figure 13:**
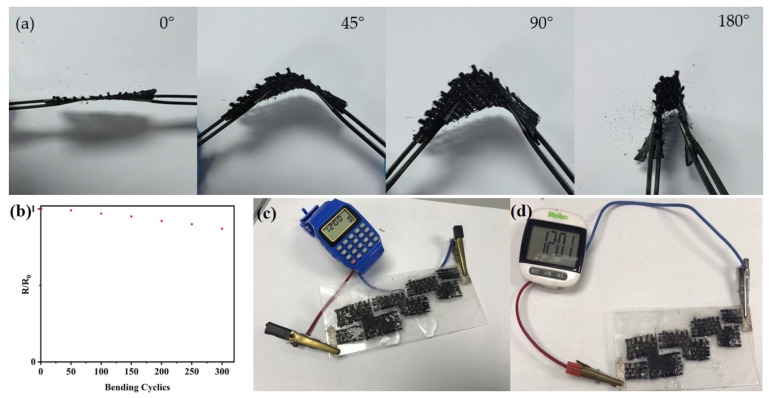
(**a**) The MnO@rGO_(0.8:1)_/C electrode under 45°, 90°, and 180° bending angles. (**b**)The electrode resistance stability photos of the four MnO@rGO_(0.8:1)_/C supercapacitors connected in series to light-up (**c**) a watch and (**d**) a thermo-hygrometer’s display screen, respectively.

Considering flexible supercapacitor applications, we evaluated the mechanical flexibility and electrode resistance stability during the deformation of the as-prepared MnO@rGO_(0.8:1)_/C electrode with the active mass loading of 5.40 mg cm^−2^, which is essential for wearable electronics. The MnO@rGO_(0.8:1)_/C electrode possesses superior mechanical flexibility, which can be restored to the original condition under 45°, 90°, and 180° bending angles, as shown in Figure 13a. Meanwhile, the electrode resistance stability is displayed in Figure 13b. We can see that the MnO@rGO_(0.8:1)_/C electrode shows less obvious deterioration in its resistance. To verify the feasibility of our MnO@rGO_(0.8:1)/_C supercapacitors for practical applications, we assembled a type of MnO@rGO/C supercapacitor system by electrically connecting four pieces of MnO@rGO_(0.8:1)_/C supercapacitors in series. As shown in Figure 13c, the integrated MnO@rGO_(0.8:1)_/C supercapacitor can provide enough power output to light an electronic watch (Figure 13a) and a thermo-hygrometer (Figure 13d).

## 4. Conclusions

In summary, we report a simple approach for the fabrication of high-performance flexible carbon fabric combined with MnO and rGO. RGO can provide a path for electron transfer, which improves the surface contact of electrode and electrolyte. In the meantime, the presence of MnO can also reduce the aggregation of graphene. The optimized MnO@rGO_(0.8:1)_/C electrodes with 5.40 mg cm^−2^ mass loadings of active material supercapacitor electrode show a high areal specific capacitance of 831.25 mF cm^−2^ and good cyclic stability of 97.2% capacitance retention after 10,000 charge–discharge cycles. The direct coating of MnO@rGO on carbon fabrics method, with excellent electrochemical properties, provides a promising electrode material in the fields of wearable energy storage supercapacitors.

## Data Availability

The raw data presented in this study are available upon request from the corresponding author.
